# The UV-B-Induced Transcription Factor HY5 Regulated Anthocyanin Biosynthesis in *Zanthoxylum bungeanum*

**DOI:** 10.3390/ijms23052651

**Published:** 2022-02-28

**Authors:** Jing Zhou, Jiaxin Meng, Shuangyu Zhang, Rufei Chi, Cheng Wang, Dongmei Wang, Houhua Li

**Affiliations:** 1 College of Landscape Architecture Sand Art, Northwest A&F University, Xianyang 712100, China; zjmmxx@nwafu.edu.cn (J.Z.); mengjiaxin26@nwafu.edu.cn (J.M.); zhangshungyu@nwafu.edu.cn (S.Z.); chirufei@nwafu.edu.cn (R.C.); 2 College of Forestry, Northwest A&F University, Xianyang 712100, China; jmcookie@nwsuaf.edu.cn

**Keywords:** *Zanthoxylum bungeanum*, anthocyanins, UV-B, *ZbHY5*, *ZbMYB113*

## Abstract

Pericarp color is an important economic characteristic of *Zanthoxylum bungeanum*. Anthocyanins are the main reason for the pericarp’s red appearance in *Z. bungeanum*. In this study, through the combined analysis of the metabolome and transcriptome, *HY5*, whose expression is highly correlated to changes in the anthocyanin content, was screened and identified. Under natural ripening conditions, the *Z. bungeanum* fruit gradually changed in color from green to red, while bagging resulted in the fruit maintaining its green color. After unbagging, the fruit gradually turned red, and the *ZbHY5* expression and anthocyanin content increased. In addition, the leaves changed from green to red after exposure to UV-B radiation, and the *ZbHY5* expression and anthocyanin content increased. The transient overexpression of *ZbHY5* deepened the redness of the *Z. bungeanum* leaves and promoted the expression of *ZbHY5* and *ZbMYB113* as well as anthocyanin accumulation. Bimolecular fluorescence complementation (BIFC) showed that there was an interaction between ZbHY5 and ZbMYB113. These results revealed that under UV-B irradiation, ZbHY5 might regulate the expression levels of the structural genes related to anthocyanin biosynthesis through combination with ZbMYB113, thereby affecting anthocyanin accumulation. This finding provides useful insights for further studies focusing on UV-B-induced anthocyanin accumulation in *Z. bungeanum*.

## 1. Introduction

*Zanthoxylum bungeanum* (Rutaceae family) has a long cultivation and application history of nearly three thousand years in China. The brilliant red pericarp color of *Z. bungeanum*, which is greatly appreciated as a traditional condiment by consumers, is due to anthocyanins, water-soluble flavonoid pigments. Anthocyanins are widely found in the flowers, stems, leaves, fruits, and other tissues of plants and are one of the main causes of plant color presentation. Additionally, anthocyanins are potentially beneficial to human health and have demonstrated anti-cancer, anti-inflammatory, anti-aging, and anti-obesity effects; improvements in brain function; and eyesight and cardiovascular protection, etc. They have also been recognized as important antioxidants [1].

Phenylalanine is the direct precursor of anthocyanins and other flavonoids. It is converted to ρ-coumaroyl CoA via the action of the phenylalanine lyase (PAL), cinnamate 4-hydroxylase (C4H) and 4-coumarate: CoA ligase (4CL). In general, anthocyanins are catalyzed from ρ-coumaroyl CoA and malonyl-CoA by a series of enzymes that are encoded by structural genes, which mainly include the early biosynthesis genes (EBGs) *CHS* (Chalcone synthase gene), *CHI* (Chalcone isomerase gene), and *F3H* (flavanone-3-hydroxylase gene), and the late biosynthesis genes (LBGs) *F3′H* (flavonoid 3′-hydroxylase gene), *F3′5′H* (flavonoid 3′,5′-hydroxylase gene), *DFR* (dihydroflavonol-4-reductase gene), *ANS* (anthocyanin synthase gene), and *UFGT* (flavonoid 3-*O*-glucosyltransferase gene) [2]. In addition, the transcription of these structural genes is controlled by regulatory factors, including by the members of protein families that contain R2R3-MYB domains, bHLH (basic helix–loop–helix) domains, and conserved WD40 repeat transcription factors (TFs) [3]. For example, MYB113, an R2R3-MYB gene, has been proven to be involved in anthocyanin synthesis in plants such as *A. thaliana* and *Pistacia chinensis* [4,5]. Additionally, bHLH35 was shown to regulate anthocyanin synthesis at high temperatures in *Solanum melongena* [6]. In recent years, other transcription factors have also been found to be involved in the anthocyanin synthesis, such as bZIP and WRKY. A recent study found that WRKY co-regulates the anthocyanin pathway synthesis with the MBW complex (the transcriptional complex of the MYB, bHLH, and WD40 proteins) [7].

Light is an important environmental factor that can regulate various plant growth and development processes, such as seed germination, morphogenesis, shade response, flowering, and senescence. Light is not only an energy source for photosynthesis, but it is also a biological stimulus that is present throughout the life cycle of plants, triggering cellular responses through specific sensory photoreceptors [8]. The current research has shown that, in many plants, the light signal transduction pathways are able to regulate anthocyanin accumulation to a certain extent [9,10]. In *Arabidopsis thaliana*, some light-induced transcription factor genes can promote anthocyanin biosynthesis, such as *MYB* (*AtMYB75* and *AtMYB90*) and *bHLH* (*AtTT8*, *AtGL3* and *AtEGL3*) [11]. In strawberry (*Fragaria×ananassa*) fruit, the transient overexpression of the light-induced *FvbHLH9* can promote the accumulation of anthocyanins, while the interference of *FvbHLH9* can reduce anthocyanin accumulation [12]. In recent years, in addition to MYB, bHLH, and WD40, some other transcription factors were also found to be involved in anthocyanin synthesis. Among them, ELONGATED HYPOCOTYL 5 (HY5) is the most critical and representative transcription factor in plant photomorphogenesis and plays a significant role in the regulation of anthocyanin biosynthesis [13]. It should be noted that the production of other flavonoids was also affected by light. *HY5* promoted flavonol accumulation through the activation of *MYB12* expression in *A. thaliana* [14].

HY5 is a basic leucine zipper (bZIP) transcription factor. The HY5 protein was shown to experience degradation in dark environments; providing UV-B prevented this degradation, *HY5* gene expression was reactivated, and HY5 protein was accumulated once again [15]. In *Arabidopsis thaliana*, it was found that HY5 can target a large quantity of light-responsive genes in vivo. This is mainly due to the typical bZIP domain of HY5, which can directly bind to the G-box (CACGTG) element or ACGT-containing elements (ACEs) of their promoters [16]. In addition, in vitro and in vivo analyses have shown that HY5 acts as a positive regulator in the anthocyanin accumulation process by directly binding to the promoters of structural genes during anthocyanin biosynthesis [17]. In addition, MYB113 was found to interact with HY5 to positively regulate anthocyanin biosynthesis in *A. thaliana* and *Malus domestica* [18].

In this study, a bZIP transcription factor ZbHY5 was isolated from *Z. bungeanum*. Bioinformatics analysis and transient gene overexpression confirmed that *ZbHY5* played a positive regulatory role in the anthocyanin biosynthesis of *Z. bungeanum*. Additionally, through BIFC, we found that there was an interaction between ZbHY5 and ZbMYB113. This study verified that *HY5* was involved in the regulation of *Z. bungeanum* anthocyanin biosynthesis, providing a theoretical reference for the light-induced anthocyanin accumulation of *Z. bungeanum*.

## 2. Results

### 2.1. Analyses of Anthocyanin Composition and Content during the Ripening of Z. bungeanum Fruit

The metabolome data showed that eight anthocyanins were detected during the four *Z. bungeanum* fruit-ripening stages: Cyanidin-3-*O*-galactoside, Cyanidin-3-*O*-glucoside (Kuromanin), Cyanidin-3-*O*-rutinoside (Keracyanin), Peonidin-3-*O*-glucoside, Delphinidin-3-*O*-(6’’-*O*-acetyl) glucoside, Pelargonidin-3-*O*-rutinoside, Pelargonidin-3,5-*O*-diglucoside, and Cyanidin-3-*O*-galactoside. Among them, the Cyanidin-3-*O*-galactoside content was the highest, and the Cyanidin-3,5-*O*-diglucoside (Cyanin) content was the second highest. Specifically, the total anthocyanin content increased throughout the four stages (Table 1).

### 2.2. Analysis of Expression Levels of Anthocyanin Biosynthesis-Related Genes during the Ripening of Z. bungeanum Fruit

The transcriptome data showed that during the *Z. bungeanum* fruit-ripening process, the *ZbHY5* and *ZbMYB113* expression levels gradually increased. The structural genes related to anthocyanin biosynthesis (*ZbCHS*, *ZbCHI*, *ZbDFR*, *ZbANS*) followed the expression pattern of these transcription factors with a short delay. The *ZbDFR* expression level at S4 was only slightly above the level at S1, while *ZbCHS*, *ZbCHI* and *ZbANS* increased 4.6-, 1.6-, and 2.9-fold, respectively. This suggested that different target genes were activated with different strengths. HY5 is a bZIP transcription factor that plays a key role in anthocyanin biosynthesis. *ZbMYB113*, whose homologous gene *AtMYB113* was also reported to be important in anthocyanin biosynthesis, presented the same change trend with *ZbHY5* (Table 2).

### 2.3. Phylogenetic Tree Analysis of HY5 and Other bZIP Family Transcription Factors in Z. bungeanum

**Figure 1:**
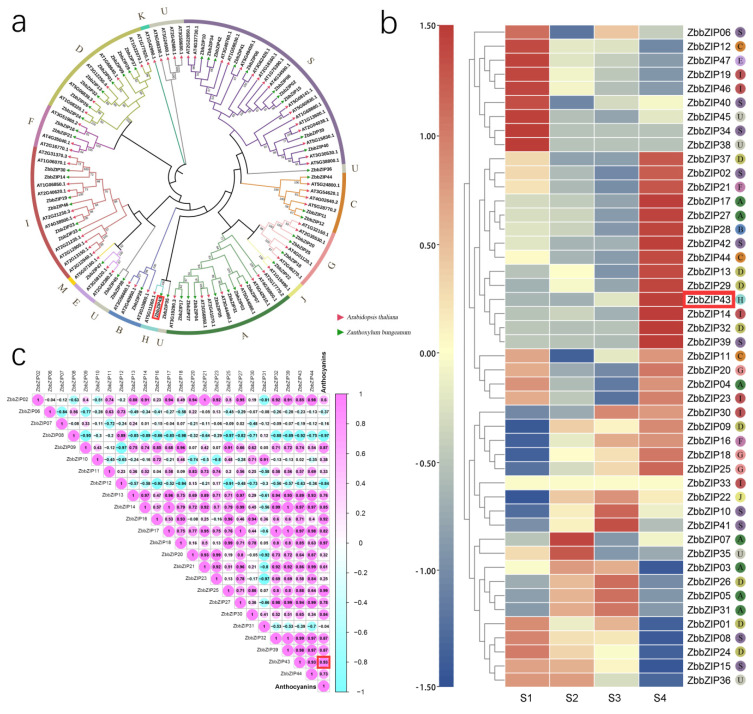
Phylogenetic tree and correlation analysis of HY5 and other bZIP family transcription factors in *Z. bungeanum*. (**a**) Phylogenetic tree of ZbbZIP transcription factor family. The phylogenetic tree was constructed using the neighbor joining (NJ) method. The ZbHY5 candidate, ZbbZIP43, is marked by the red box. (**b**) Heat map of bZIP transcription factor gene expression in *Z. bungeanum* fruits at different growth and development stages. *ZbbZIP43* is marked by the red box. S1–S4 represent the four gradual ripening stages of *Z. bungeanum* fruit: S1 (green stage), S2 (light red stage), S3 (red stage), and S4 (deep red stage). (**c**) Heat map representing the correlation between bZIP transcription factor gene expression and the anthocyanin content in the different growth and development stages of *Z. bungeanum* fruit. The number in the box represents the correlation between the abscissa and the ordinate. The positive value represents a positive correlation, the negative value represents a negative correlation, and the larger the absolute value of the number is, the stronger the correlation is. The 0.93 marked by the red box shows that there was a positive correlation between the anthocyanin content and *ZbbZIP43* expression, and the correlation coefficient was 0.93.

Through a keyword search, local blastp alignment, a local hmmer search, and the deletion of sequences whose domains were incomplete, 47 target sequences were finally obtained as bZIP family protein sequences of *Z. bungeanum*, which were named ZbbZIP01—47 according to their ID numbers in the transcriptome. Wolfgang et al. (2018) updated the classification method of the bZIP transcription factor family in the model plant *Arabidopsis thaliana* in 2018 [19]. They divided the bZIP transcription factor of *Arabidopsis thaliana* into 12 subfamilies, which were labeled from A to K, and S. Zhang et al. (2011) studied *Arabidopsis thaliana* and found that HY5 and HYH in the H subfamily were involved in anthocyanin synthesis [20]. Through the analysis of the phylogenetic relationship with *Arabidopsis thaliana*, it was found that there was only one H subfamily member in *Z. bungeanum*—ZbHY5 (ZbbZIP43) (Figure 1a). The analysis of the differential expression patterns showed that the expression level of *ZbHY5* was relatively high throughout all stages and showed a gradual upward trend as the *Z. bungeanum* fruit grew and developed (Figure 1b). The correlation analysis heat map showed that the expression level of *ZbHY5* was positively correlated with the total anthocyanin content and that the correlation coefficient was as high as 0.93 (Figure 1c). These results proved that we successfully screened *ZbHY5*, the key transcription factor gene involved in anthocyanin synthesis, from the bZIP family of *Z. bungeanum*.

### 2.4. Clone and Identification of HY5 in Z. bungeanum

**Figure 2:**
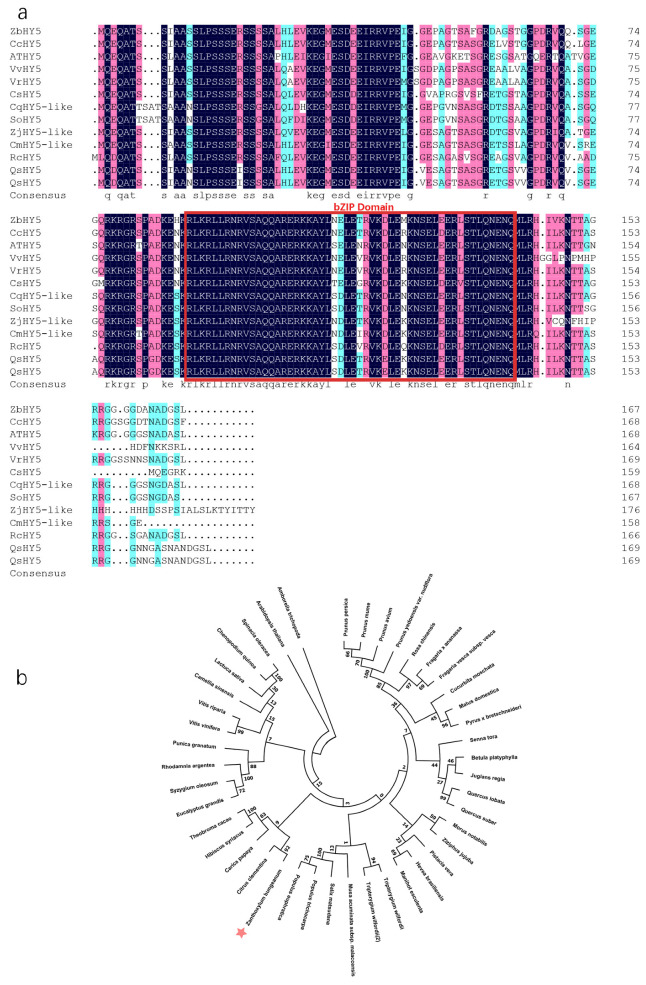
Sequence alignment and phylogenetic analysis of ZbHY5. (**a**) Protein alignment of ZbHY5 and its homologs, where the bZIP domain is marked with a red box. The phylogenetic tree was constructed using the neighbor joining (NJ) method. (**b**) Phylogenetic analysis of ZbHY5 and 42 other plants. *Zanthoxylum bungeanum* (ZbHY5) is denoted by a red asterisk. *Arabidopsis thaliana*, BDQV01000026.1; *Betula platyphylla*, AHY20043.1; *Citrus clementina*, XP_006450470.1; *Cucurbita moschata*, XP_022962152.1; *Carica papaya*, XP_021891410.1; *Chenopodium quinoa*, XP_021758181.1; *Camellia sinensis*, XP_028120847.1; *Eucalyptus grandis*, XP_010048982.1; *Fragaria x ananassa*, AKG58815.1; *Fragaria vesca* subsp. *Vesca*, XP_004291469.1; *Hevea brasiliensis*, XP_021670969.1; *Hibiscus syriacus*, XP_039039624.1; *Juglans regia*, XP_035548793.1; *Lactuca sativa*, XP_023759627.1; *Musa acuminata* subsp. *Malaccensis*, XP_009381123.1; *Malus domestica*, NP_001280752.1; *Manihot esculenta*, XP_021597568.1; *Morus notabilis*, XP_010110356.2; *Prunus avium*, XP_021827650.1; *Pyrus x bretschneideri*, XP_009348603.1; *Populus euphratica*, XP_011039711.1; *Punica granatum*, XP_031407456.1; *Prunus mume*, XP_008219477.1; *Prunus persica*, XP_020411091.1; *Populus trichocarpa*, XP_002308656.1; *Pistacia vera*, XP_031286333.1; *Prunus yedoensis* var. *Nudiflora*, PQM36064.1; *Quercus lobata*, XP_030951444.1; *Quercus suber*, XP_023893213.1; *Rhodamnia argentea*, XP_030548349.1; *Rosa chinensis*, XP_024165510.1; *Salix matsudana*, QEH62726.1; *Spinacia oleracea*, XP_021837612.1; *Senna tora*, KAF7817238.1; *Syzygium oleosum*, XP_030440254.1; *Theobroma cacao*, XP_007013841.2; *Tripterygium wilfordii*, KAF5743305.1; *Tripterygium wilfordii*, XP_038698890.1; *Vitis riparia*, XP_034682512.1; *Vitis vinifera*, XP_010648648.1; *Ziziphus jujuba*, XP_024925020.1.

In *Arabidopsis thaliana* and some other plants, *HY5* plays an important role in anthocyanin biosynthesis [16,21]. To verify the function of *HY5* in *Z. bungeanum*, the *HY5* gene was cloned from *Z. bungeanum* leaves. The gene sequencing results showed that the *HY5* had a 504-bp ORF, which encoded a protein containing 167 amino acids. Multiple sequence alignments showed that the C-terminus of *HY5* contained a bZIP domain (amino acids 90–141) (Figure 2a). This was designated as *ZbHY5*.

The phylogenetic tree of HY5 in *Z. bungeanum* and the other 42 plants showed that ZbHY5 had the highest homology with CcHY5 (*Citrus clementina*) (Figure 2b). Loyola et al. proved that HY5 plays an important role in the transmission of light signals and the regulation of anthocyanin accumulation in grapes [22]. Huang et al. found that HY5 is involved in anthocyanin synthesis in some citrus plants, such as in blood orange and purple pummelo. These results revealed that *ZbHY5* may be related to anthocyanin regulation in *Z. bungeanum* and provided a basis for further research [23].

### 2.5. Bagging Inhibited Anthocyanin Synthesis in Z. bungeanum Fruit

**Figure 3:**
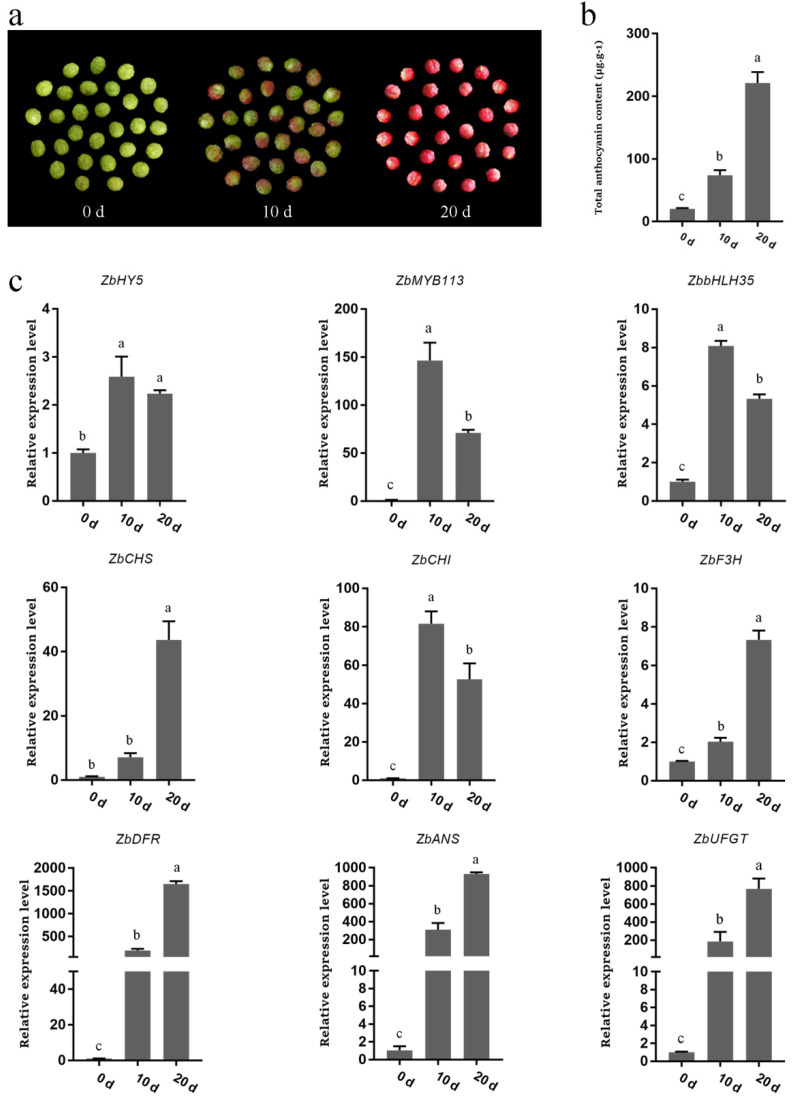
Total anthocyanin content and gene expression changes after the unbagging of *Z. bungeanum* fruit. (**a**) Pictures of *Z.* *bungeanum* fruit at 0 days (0 d), 10 days (10 d), and 20 days (20 d) after unbagging. (**b**) Total anthocyanin content in 0 d, 10 d, and 20 d fruit. (**c**) The expression of the transcription factor genes (*ZbHY5*, *ZbMYB113*, *ZbbHLH35*) and structural genes (*ZbCHS*, *ZbCHI*, *ZbF3H*, *ZbDFR*, *ZbANS*, *ZbUFGT*) related to anthocyanin synthesis in 0 d, 10 d, and 20 d fruit. Different lowercase letters indicate significant differences among treatments according to one-way ANOVA test (*p* < 0.05).

In the fruit that had been taken out of the bags at 0 days (0 d), 10 days (10 d), and 20 days (20 d), the color of the pericarp gradually changed from green to red. Bagging, on the one hand, delayed the pericarp coloring process and resulted in the color of the bagged fruit being significantly lighter than that of the fruit exposed to light (Figure 3a).

The results of the total anthocyanin content determination were consistent with the phenotypic changes that we observed. The total anthocyanin content in the pericarp showed a gradual upward trend from 0 d to 10 d, and it then increased rapidly in the 20 d fruit (Figure 3b). This suggests that anthocyanin accumulation is delayed to a certain extent after bagging and that the anthocyanin content would eventually be reduced.

Compared to 0 d, the expression levels of the transcription factor genes (*ZbHY5*, *ZbMYB113*, *ZbbHLH35*) and the structural genes (*ZbCHS*, *ZbCHI*, *ZbF3H*, *ZbDFR*, *ZbANS*, *ZbUFGT*) related to anthocyanin synthesis in the 10 d and 20 d fruits increased significantly. However, compared to 10 d, only the expression levels of *ZbCHS*, *ZbF3H*, *ZbDFR, ZbANS,* and *ZbUFGT* increased in the 20 d fruit, while the expression levels of the other genes (*ZbHY5*, *ZbMYB113*, *ZbbHLH35,* and *ZbCHI*) decreased slightly (Figure 3c). This might be due to the sudden exposure to light after long-term shading increasing the expression levels of genes in response to sharp increases in the amount of light and allowing the genes to achieve their maximum expression in a short time, exceeding the expression levels of these genes in the 20 d samples. These results revealed that light could significantly promote anthocyanin accumulation in *Z. bungeanum* by regulating the expression of related genes.

### 2.6. UV-B Irradiation Promoted Anthocyanin Synthesis in Z. bungeanum Leaves

**Figure 4:**
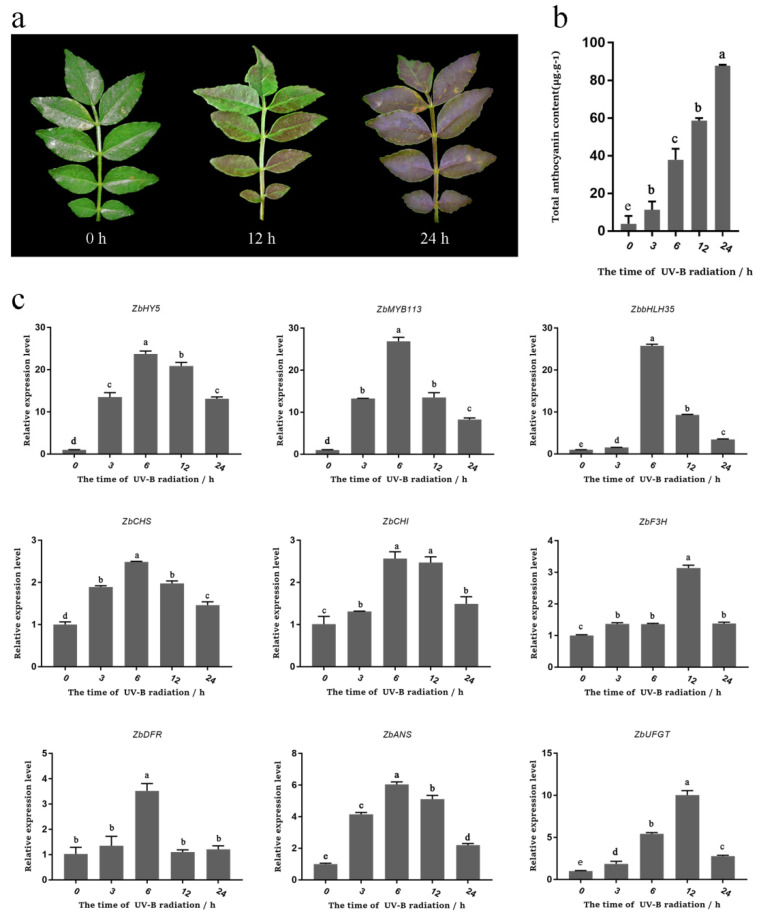
Changes in the total anthocyanin content and gene expression under UV-B irradiation in *Z. bungeanum* leaves. (**a**) Pictures of the *Z. bungeanum* leaves under UV-B irradiation after 0 h, 12 h, and 24 h. (**b**) The total anthocyanin content in the leaves after UV-B irradiation for 0 h, 3 h, 6 h, 12 h, and 24 h. (**c**) The expression of the transcription factor genes (*ZbHY5*, *ZbMYB113*, *ZbbHLH35*) and structural genes (*ZbCHS*, *ZbCHI*, *ZbF3H*, *ZbDFR*, *ZbANS*, *ZbUFGT*) related to anthocyanin synthesis in the leaves after UV-B irradiation for 0 h, 3 h, 6 h, 12 h, and 24 h. Different lowercase letters indicate significant differences among treatments according to one-way ANOVA test (*p* < 0.05).

After UV-B irradiation, the leaves of *Z. bungeanum* changed from green to red. No obvious changes were observed from 0 to 6 h, but the leaves gradually turned red after 12 h (Figure 4a). Consistent with the phenotypic changes, total anthocyanin content in the leaves gradually increased from 0 to 24 h (Figure 4b). Interestingly, the qRT-PCR results showed that the expression levels of *ZbHY5*, *ZbMYB113*, *ZbbHLH35, ZbCHS*, *ZbCHI, ZbF3H, ZbDFR, ZbANS,* and *ZbUFGT* presented increasing and then decreasing trends. Among these genes, the expression levels of *ZbHY5*, *ZbMYB113*, *ZbCHS*, *ZbCHI*, *ZbDFR,* and *ZbANS* reached their maximum levels at 6 h, while the expression levels of *ZbF3H* and *ZbUFGT* reached their maximum levels at 12 h (Figure 4c). These results indicated that UV-B promotes anthocyanin accumulation in the leaves of *Z. bungeanum* by controlling the expression of related genes.

### 2.7. Transient Overexpression of ZbHY5 Promoted Anthocyanin Synthesis of Z. bungeanum Leaves

**Figure 5:**
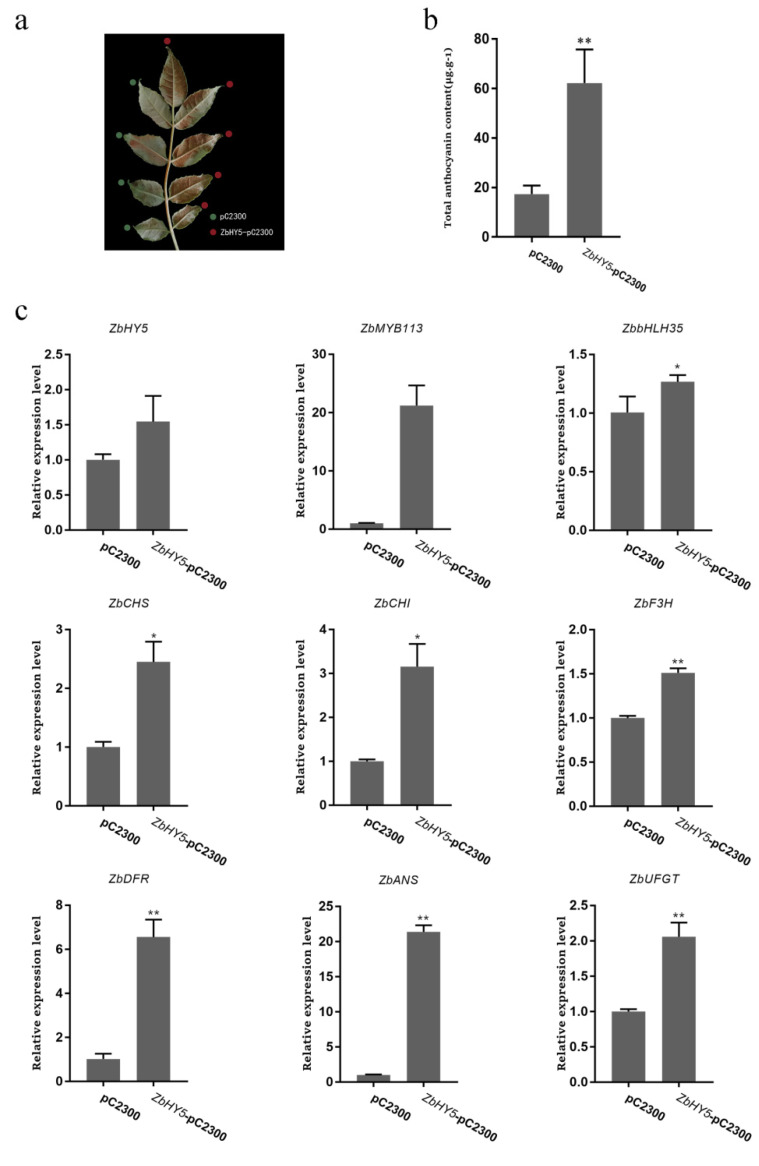
(**a**) Pictures of *ZbHY5* gene overexpression phenotype changes in *Z. bungeanum* leaves. The green marks represent the control (pC2300) leaves, and the red marks represent the experimental group (*ZbHY5*-pC2300) leaves. (**b**) Determination of total anthocyanin content in *ZbHY5* gene overexpression in *Z. bungeanum* leaves. (**c**) Quantitative analysis of genes related to *ZbHY5* gene overexpression in the leaves. * *p* < 0.05, ** *p* < 0.01 using the *t*-test.

Transient *ZbHY5* overexpression was performed through infecting *Z. bungeanum* leaves with *Agrobacterium tumefaciens* containing empty pC2300 as the control and with *Agrobacterium tumefaciens* containing *ZbHY5*-pC2300, which served as the experimental group. After infection, the leaves were cultured in the dark for 24 h and then under UV-B irradiation for 18 h. The leaves overexpressing *ZbHY5* turned dark red, while the control leaves only showed a slight red coloration (Figure 5a). The total anthocyanin content in the leaves overexpressing *ZbHY5* was significantly higher than that in the control leaves, with a 3.59-fold difference (Figure 5b). Additionally, further quantitative analysis found that the expression levels of *ZbMYB113*, *ZbbHLH35*, *ZbCHS*, *ZbCHI*, *ZbF3H*, *ZbDFR*, *ZbANS,* and *ZbUFGT,* which were related to anthocyanin biosynthesis in the leaves overexpressing *ZbHY5*, were also increased compared to the control 21.19-, 1.26-, 2.44-, 3.15-, 1.51-, 6.43-, 18.16-, and 2.06-fold, respectively (Figure 5c).

These results suggested that *ZbHY5* could promote anthocyanin synthesis by inducing the expression of the key genes related to anthocyanin biosynthesis, leading to the accumulation of anthocyanin in the leaves of *Z. bungeanum*.

### 2.8. ZbHY5 Can Interact with ZbMYB113 In Vivo

**Figure 6:**
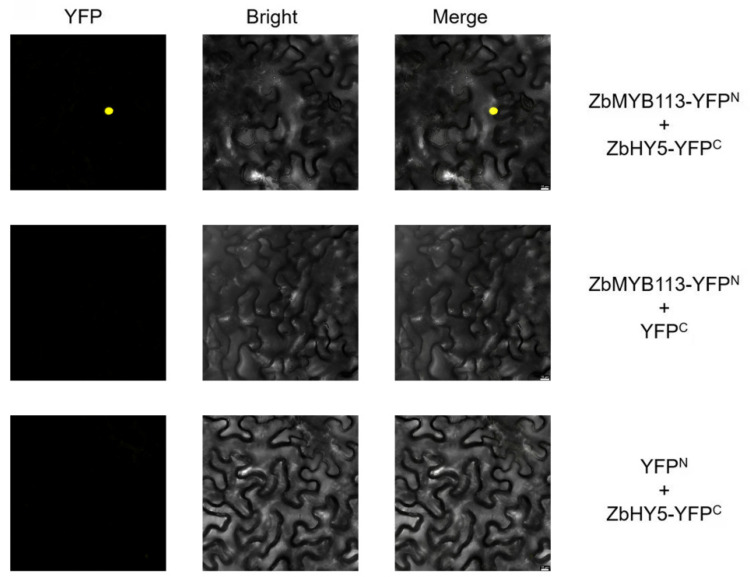
The BiFC assay was carried out to verify the interaction between ZbHY5 and ZbMYB113. Bars, 10 µm.

In order to further study the anthocyanin synthesis regulation mechanism, the BiFC assay was performed to verify whether ZbHY5 could bind to ZbMYB113 to form a protein complex in vivo. Observation using a confocal laser scanning microscope revealed that only yellow fluorescence is observed in the nucleus of tobacco leaf cells when ZbHY5-YFP^C^ and ZbMYB113-YFP^N^ are co-expressed. In contrast, there was no signal in the combinations of *ZbMYB113*-YFP^N^ + YFP^C^ or YFP^N^ + *ZbHY5*-YFP^C^ (Figure 6). Therefore, the BiFC assay confirmed that ZbHY5 can interact with ZbMYB113.

## 3. Discussion

For most plants, UV-B, the middle ultraviolet light (λ = 280–315 nm), is an important factor for anthocyanin accumulation [24]. UV-B can promote anthocyanin accumulation because it promotes the expression of structural genes and regulatory genes in the anthocyanin biosynthesis pathway [11]. HY5, a bZIP transcription factor that participates in anthocyanin biosynthesis, is a downstream component of the light signaling pathway and is responsive to UV-B [25,26,27]. In *Arabidopsis thaliana* [14], *Solanum lycopersicum* [28], and blood orange (*Citrus*) [23], the HY5 protein was confirmed to participate in the anthocyanin synthesis process. In this experiment, the *Z. bungeanum* fruit gradually changed from green to red under natural growth conditions because of anthocyanin accumulation, and the transcriptome data showed that the *ZbHY5* expression level gradually increased. On the contrary, bagging blocked the fruit’s reddening process, inhibited the expression of *ZbHY5*, and lessened the anthocyanin accumulation in *Z. bungeanum* fruit. In addition, under UV-B radiation, the leaves turned from green to red, from the *ZbHY5* expression, and the anthocyanin content increased in the *Z. bungeanum* leaves. The transient overexpression of *ZbHY5* in the leaves of *Z. bungeanum* caused significantly red coloration after 18 h of UV-B irradiation. The total anthocyanin content and the *ZbHY5* expression levels increased significantly in the leaves overexpressing *ZbHY5*. These results verified that under UV-B irradiation, *ZbHY5* played a key role in anthocyanin accumulation in *Z. bungeanum*.

HY5 can not only directly regulate the expression of the early and late structural genes in the downstream anthocyanin synthesis pathway [29], but it can also interact with other transcription factor proteins to indirectly regulate the late structural genes [30,31]. In *Arabidopsis thaliana*, HY5 bound itself directly to the PAP1 in vivo to regulate anthocyanin biosynthesis [32]. In *Pyrus pyrifolia*, HY5 physically interacted with MYB10 to positively regulate anthocyanin biosynthesis [18,33]. In this study, the transcription factor *ZbMYB113* was screened and confirmed to be one of the R2R3-MYB activators involved in anthocyanin biosynthesis from *Z. bungeanum*. The transcriptome data showed that during the reddening of the *Z. bungeanum* fruit, *ZbHY5* and *ZbMYB113* expression levels gradually increased, and there was a high correlation between their expression levels. Bagging restrained the red process and the *ZbHY5* and *ZbMYB113* expression levels in *Z. bungeanum* fruit. Moreover, UV-B radiation made the leaves turn from green to red and promoted *ZbHY5* and *ZbMYB113* expression in *Z. bungeanum*. Furthermore, the overexpression of *ZbHY5* deepened the redness of the leaves and enhanced *ZbMYB113* expression. Through the BiFC assay, we found that ZbMYB113 can interact with ZbHY5 in vivo. These results suggested that the anthocyanin synthesis pathway in *Z. bungeanum* is co-regulated by ZbMYB113 and ZbHY5. In addition, HY5 also regulated the flavonol biosynthesis in response to UV-B [14]. This suggested that the role of UV-B-induced HY5 in flavonoid synthesis deserves further research.

Studies have found that HY5 regulates anthocyanin biosynthesis in two ways. On the one hand, it directly binds and induces the expression of some MYB transcription factor genes. On the other hand, it co-induces the expression of anthocyanin biosynthetic enzyme genes by interacting with other MYB factors [13]. In this study we focused on the interaction between ZbHY5 and ZbMYB113 and the result of BIFC also demonstrated the interaction between them. Furthermore, in the overexpression experiments, the expression level of *ZbMYB113* increased 20-fold, far exceeding that of *ZbHY5*. This suggests that in addition to the interaction, ZbHY5 may also be a regulator of ZbMYB113. This requires further research. MYB4 is a repressor of the anthocyanin synthesis pathway in response to UV-B. This may be the reason why the expression of some genes such as DFR decreased after some time. This is a good direction for the follow-up study of the UV-B-induced anthocyanin synthesis pathway.

## 4. Materials and Methods

### 4.1. De Novo Assembly and Unigene Annotation

All of the assembled unigenes were annotated by a sequence similarity search using BLAST (http://blast.ncbi.nlm.nih.gov/Blast.cgi) (accessed on 13 September 2019) with an e-value ≤ 10^−5^ against other databases, including NR (ftp.ncbi.nlm.nih.gov/blast/db/FASTA/nr.gz) (accessed on 20 September 2019), KOG, COG, eggNOG, KEGG, GO, and SwissProt [34]. Additionally, the predicted amino acid sequences of the unigenes were also mapped against the Pfam database using HMMER software (version 3.2.1) with an e-value ≤ 10^−10^ [35,36]. Finally, the annotated information for the unigenes was obtained. Additionally, the automatic annotation of the flavonoid biosynthesis genes and the MYBs were used in this study [37,38].

### 4.2. Metabonomic Analysis and Transcriptome Analysis

*Z. bungeanum* fruit was collected from eight-year-old trees grown in the experimental station of Northwest A&F University, Shaanxi, Yangling, China. Fruit in the four stages of growth, S1 (green stage), S2 (light red stage), S3 (red stage), and S4 (deep red stage), were collected during the ripening process, and each sample had three biological replicates [39]. Fruit from each stage were divided into two groups: one for metabonomic flavonoid analysis and the other for transcriptome sequencing.

### 4.3. The Treatment of Bagging Fruit

About a month before maturity, the *Z. bungeanum* fruits were bagged in the field on 17 May 2020. After 20 d (6 June 2020), some of the bagged fruit (0 d) were collected for return to the laboratory, and the remaining bagged fruit were removed from the bags and collected after being exposed to natural light for 10 d (16 June 2020) and 20 d (26 June 2020) [18]. All the fruit came from different branches of the sunny side of the same tree, and they were all at a height of about 1.5 m, which was convenient for bagging and picking. All the fruit were collected at 9:00–10:00 a.m. The samples were stored in a refrigerator at –80 °C after being quick-frozen in liquid nitrogen for subsequent experiments.

### 4.4. The Treatment of UV-B Irradiation for Leaves

The leaves were collected from annual hydroponic seedlings of *Z. bungeanum* grown in a greenhouse. The annual *Z. bungeanum* seedlings were grown under continuous irradiation in Philips TL20W/01RS narrowband UV-B tubes (1.5 mmol m^−2^ s^−1^; measured with a VLX-3W Ultraviolet Light Meter equipped with a CX-312 sensor, Vilber Lourmat, Marne-laVallée, France). All the irradiated seedlings were darkened for 24 h in advance [40]. Then, the leaves were collected after 0 h, 3 h, 6 h, 12 h, and 24 h of irradiation [18]. The samples were stored in a refrigerator at –80 °C after being quick-frozen in liquid nitrogen for subsequent experiments.

### 4.5. Bioinformatics Analysis of ZbbZIP Transcription Factor Family

#### 4.5.1. Excavation and Identification of ZbbZIP Transcription Factor Family Members

The transcriptional data related to anthocyanin biosynthesis in *Z. bungeanum* were screened from our previous transcriptome. The keywords “bZIP” and “basic leucine zipper” were searched directly in the transcriptome database, the two search results were summarized, and the repetitive sequences were deleted. The bZIP family protein sequence of the model plant *Arabidopsis thaliana* was downloaded from the TAIR database (http://web.arabidopsis.org) (accessed on 2 December 2020) as the query sequence, the blastp alignment (e-value set to 1e-5) was performed in the *Z. bungeanum* transcriptome data through the local BLAST+ software, and the repetitive sequences were deleted. Hidden Markov Models (HMMs) (PF00170, PF03131, and PF07716) of the conserved domains of the bZIP family were downloaded from the Pfam database (http://pfam.xfam.org) (accessed on 5 December 2020). The HMMs of the three bZIP domains were used as the query sequence to search the *Z. bungeanum* transcriptome data using the local HMMER 3.0 software; sequences whose e-values were less than 1e-5 were kept, and the repetitive sequences were deleted. The common items in the sequences obtained by the above three methods were taken as the candidate protein sequences of the bZIP family in *Z. bungeanum* [41,42].

The online software InterPro (http://www.ebi.ac.uk/interpro/) (accessed on 10 December 2020) was used to identify and analyze the conserved domain of the candidate protein sequence, and the protein sequences not containing the bZIP domain were removed. The local MAFFT software was used to align all of the bZIP domain sequences and to manually check and delete the sequences with incomplete domains [43,44].

#### 4.5.2. Construction of Phylogenetic Tree

The local MAFFT software was used to perform multiple sequence alignments on the bZIP protein sequences of *Z. bungeanum* and *Arabidopsis thaliana*. The alignment results were imported into MEGA 7, and the neighbor joining (NJ) method was used to construct a phylogenetic tree. The parameter settings were as follows: bootstrap method 1000; P-distance model; partial deletion; cutoff 50. Then, the online software ITOL was used to beautify the phylogenetic tree [41].

*ZbHY5* was cloned, and the nucleotide sequence and amino acid sequence of ZbHY5 were obtained by gene sequencing. The amino acid sequences of the HY5 transcription factor in 40 kinds of plants (such as *Arabidopsis thaliana*, *Citrus clementina*, *Malus domestica*, etc.) were obtained from the NCBI database using BLASTP technology. MEGA 7 software was used to construct a phylogenetic tree [18].

#### 4.5.3. Differential Expression Pattern Analysis of ZbbZIP Transcription Factor Family Genes

The FPKM values of the *ZbbZIP* transcription factor family genes were taken from the transcriptome, the logarithmic calculation method was used to perform hierarchical cluster analysis on the expression data of the *ZbbZIP* family genes, and Tbtools software was used to draw the expression heat map [41].

#### 4.5.4. Correlation Analysis of Anthocyanin Content and ZbbZIP Transcription Factor Family Gene Expression

The FPKM values (greater than 20) of the *ZbbZIP* transcription factor family genes were taken in the transcriptome, and the total anthocyanin contents of the four growth and development stages in *Z. bungeanum* were taken from metabolome, and the local software R 3.6.1 was used to draw the correlation analysis heat map.

### 4.6. The Extraction and Measurement of Total Anthocyanins

An amount of 0.15 g freeze-dried *Z. bungeanum* leaves or pericarps was weighed, quickly ground into powders in liquid nitrogen and soaked in 3 mL extraction solution (the volume ratio of methanol and hydrochloric acid was 97:3). After the samples were shaken and mixed, they were incubated in the refrigerator at 4 °C in the dark for 48 h and subsequently shaken and mixed once every 12 h. Then, the mixed samples were ultrasonically extracted for 20 min and centrifuged at 6000 rpm for 10 min, and the supernatant was collected. Finally, with the extraction solution as a blank control, the absorbance of each sample at 530 and 657 nm was measured using an ultraviolet spectrophotometer. The total anthocyanin content (μg·g^−1^) of each sample was calculated according to the following formula: (A530-0.25 × A657) × 449.2 × 3 × 100/(26,900 × 0.15) [45].

### 4.7. Extraction of Total RNA and Real-Time Fluorescence Quantitative PCR (qRT-PCR) Analysis

The total RNA was extracted from the pericarps and leaves of *Z. bungeanum* using the Plant RNA Kit (Omega, R6827-01, Norcross, GA, USA). Then, 1000 ng RNA was used as the template to reverse-transcribe to the first-strand cDNA using the TransScript^®^ One-Step gDNA Removal and cDNA Synthesis SuperMix Kit (TransGen, AE311-03, Beijing, China). Three biological replicates were set for each treatment. The Hieff qPCR SYBR Green Master Mix kit (High Rox) (Yeasen, 11203ES08, Shanghai, China) was used for the real-time fluorescent quantitative PCR (qRT-PCR) reaction. *ZbUBQ* was selected as the internal reference gene, and the cDNA obtained by reverse transcription was diluted five times and used as a template [46]. The primers for all of genes were designed using the Oligo 6 software and synthesized by Beijing Aoke Dingsheng Biotechnology Co., Ltd. All of the primers were used after being diluted 10 times. The reaction program was as follows: 95 °C pre-denaturation for 5 min, 95 °C for 10 s, 58 °C for 20 s, 72 °C for 30 s, 40 cycles. Each sample was set for three technical repetitions, and the results were analyzed using the 2^−ΔΔCT^ method [47]. Genes such as *ZbMYB113* and *ZbbHLH35* were all identified by BLAST and phylogenetic tree analysis for qRT-PCR.

### 4.8. Construction of Transient Gene Overexpression Vector

The specific primers were designed and synthesized for transient gene overexpression. cDNA that had been reverse-transcribed from the RNA extracted from *Z. bungeanum* leaves exposed to UV-B for 6 h was used as a template. A 504 bp full-length *ZbHY5* gene was obtained by PCR amplification using the Takara high-fidelity enzyme Primmer Star. The PCR program was set to pre-denaturation at 94 °C for 3 min; denaturation at 98 °C for 10 s; annealing at 57 °C for 15 s; extension at 72 °C for 10 s and 40 cycles; and extension at 72 °C for 10 min. The target fragment was recovered. After adding the A tail, it was ligated to the cloning vector pMD19-T overnight at 4 °C to obtain the recombinant cloning vector *ZbHY5*-pMD19-T. The recombinant cloning vector was transferred into *E. coli* DH5α competent cells and was used to extract the plasmid after verification through sequencing. At the same time, the pCambia2300 (referred to as pC2300) expression vector plasmid was extracted in the laboratory. Kpn1 and Sal 1 were used to double digest the *ZbHY5*-pMD19-T and pC2300 plasmids, and the T4 ligase was used to insert *ZbHY5* into pC2300 to obtain a recombinant *ZbHY5*-pC2300 gene overexpression vector. The recombinant expression vector was transferred into *E. coli* DH5α transcompetent cells. After verification, it was transferred to competent *Agrobacterium tumefaciens* GV3101 cells and stored in a refrigerator at –80 °C for use [48].

### 4.9. Transient Gene Overexpression Assay in Z. bungeanum Leaves

An amount of 500 μL of both the *Agrobacterium tumefaciens* pC2300 and *ZbHY5*-pC2300 broths were inoculated into 1 mL of LB liquid medium (100 μL kanamycin of 50 mg·μL^−1^ per 100 mL of LB medium) and activated in a shaker at 28 °C for 12 h. An amount of 1 mL activated bacterial solution was transferred to 20 mL of LB liquid medium (containing kanamycin) and incubated on a shaker at 28 °C for 12 h to ensure that the OD_600_ of the bacterial solution reached approximately 2.0. The bacterial solution was centrifuged at 5000 rpm at 4 °C for 5 min, the bacterial cells were collected and resuspended in the resuspension solution (each 1 L resuspension solution contained 40 mL 250 mM MES (2-morpholinoethanesulfonic acid) solution, 100 μL 10 mM MgCl2 solution, and 3 mL 50 mM AS (acetosyringone) solution). The OD_600_ of the resuspended bacterial solution was calculated and adjusted to about 1.0. The resuspended bacterial solution could be used for infection after being shaken in a shaker at 28 °C at 100 rpm for 3 h in the dark [48].

A disposable sterile syringe (with the needle removed) was used to inject the main leaf vein of the dorsum of the leaf for infection. After infection, the plants were placed in a light incubator for 24 h in low temperature and dark (temperature 10 °C; humidity 75%; light intensity 0%) conditions. UV-B light was used for induction (temperature 22 °C; humidity 75%; illuminance 0%). The leaves were collected after 18 h of induction, quickly frozen in liquid nitrogen, and stored in a refrigerator at –80 °C until their next use.

### 4.10. Bimolecular Fluorescence Complementation (BiFC) Assay

The *ZbMYB113* gene was cloned with the specific primers. The full-length *ZbHY5* and *ZbMYB113* gene CDSs were, respectively, inserted into the C-terminal and the N-terminal fragment of YFP to obtain ZbHY5-YFP^C^ and ZbMYB113-YFP^N^. The recombinant and empty plasmids were transformed into *Agrobacterium tumefaciens* GV3101 cells using the freeze–thaw method. The *Agrobacterium tumefaciens* cells were infiltrated into tobacco epidermal cells with a needleless syringe. After 48 h, the YFP fluorescence signals were detected by a confocal laser scanning microscope [49].

## 5. Conclusions

The pericarp color greatly affects *Z. bungeanum* fruit quality. On the basis of the current results and those from previous studies, it can be concluded that *ZbHY5* regulates the anthocyanin biosynthesis process through activating the transcription of related genes in response to light or UV-B irradiation. In addition, the ZbHY5 regulation process likely depends on interaction with ZbMYB113. Proper spacing between fruit trees can improve the utilization of light energy, thereby enhancing the expression level of the *ZbHY5* gene. This finding suggests that high-quality fruit with a high anthocyanin content can be obtained by optimizing the spacing of fruit trees. In a word, this research provides evidence for the further investigation of the UV-B regulation of anthocyanin accumulation in *Z. bungeanum*.

## Figures and Tables

**Table 1:** Anthocyanin composition and content during the ripening of *Z. bungeanum* fruit.

Anthocyanins	S1	S2	S3	S4
Cyanidin-3-*O*-galactoside	(1.57 ± 0.02) × 10^7^ d	(1.06 ± 0.02) × 10^8^ c	(9.07 ± 0.29) × 10^7^ b	(1.25 ± 0.02) × 10^8^ a
Cyanidin-3-*O*-glucoside (Kuromanin)	(1.71 ± 0.04) × 10^6^ d	(9.87 ± 0.4) × 10^6^ c	(2.85 ± 0.01) × 10^7^ b	(6.18 ± 0.22) × 10^7^ a
Cyanidin-3-*O*-rutinoside (Keracyanin)	(6.27 ± 0.13) × 10^5^ a	(3.96 ± 0.04) × 10^5^ b	(3.12 ± 0.02) × 10^5^ c	(3.33 ± 0.06) × 10^5^ c
Peonidin-3-*O*-glucoside	(1.92 ± 0.19) × 10^3^ a	(3.42 ± 0.54) × 10^3^ a	(3.24 ± 1.70) × 10^3^ a	(1.81 ± 0.34) × 10^3^ a
Delphinidin-3-*O*-(6’’-*O*-acetyl) glucoside	(1.12 ± 0.03) × 10^6^ a	(7.90 ± 0.20) × 10^5^ b	(4.58 ± 0.19) × 10^5^ c	(3.50 ± 0.65) × 10^5^ c
Pelargonidin-3-*O*-rutinoside	(2.19 ± 0.04) × 10^5^ a	(1.41 ± 0.02) × 10^5^ b	(1.00 ± 0.03) × 10^5^ c	(8.11 ± 0.38) × 10^4^ d
Pelargonidin-3,5-*O*-diglucoside	(8.01 ± 0.45) × 10^4^ a	(3.33 ± 0.02) × 10^4^ b	(2.37 ± 0.51) × 10^4^ c	(3.76 ± 0.43) × 10^4^ b
Cyanidin-3,5-*O*-diglucoside (Cyanin)	(1.71 ± 0.02) × 10^7^ d	(1.15 ± 0.01) × 10^8^ b	(9.46 ± 0.17) × 10^7^ c	(1.36 ± 0.03) × 10^8^ a
Total anthocyanins	(3.65 ± 0.03) × 10^7^ d	(2.32 ± 0.03) × 10^8^ c	(2.15 ± 0.05) × 10^8^ b	(3.23 ± 0.03) × 10^8^ a

S1–S4 represent the four gradual ripening stages of *Z. bungeanum* fruit: S1 (green stage), S2 (light red stage), S3 (red stage), and S4 (deep red stage). Different lowercase letters indicate significant differences among treatments according to one-way ANOVA test (*p* < 0.05).

**Table 2:** Expression levels of anthocyanin biosynthesis-related genes during the ripening of *Z. bungeanum* fruit.

Gene Name	S1	S2	S3	S4
*ZbHY5*	66.33 ± 1.23 b	153.74 ± 6.13 a	172.40 ± 14.95 a	189.11 ± 2.17 a
*ZbMYB113*	1.83 ± 0.65 c	4.45 ± 1.21 c	19.71 ± 0.85 b	57.59 ± 2.73 a
*ZbbHLH35*	20.65 ± 1.02 b	41.08 ± 3.93 a	25.51 ± 1.24 b	29.41 ± 2.65 b
*ZbCHS*	160.66 ± 2.56 b	147.64 ± 3.25 b	435.95 ± 2.47 b	746.46 ± 34.78 a
*ZbCHI*	69.74 ± 1.84 c	45.62 ± 2.92 d	104.60 ± 3.88 b	115.01 ± 2.12 a
*ZbF3H*	465.99 ± 6.53 bc	439.16 ± 5.81 c	483.21 ± 12.77 b	314.87 ± 13.38 a
*ZbDFR*	46.28 ± 0.50 b	10.30 ± 0.66 d	20.39 ± 0.16 c	52.18 ± 1.77 a
*ZbANS*	52.82 ± 4.49 c	41.63 ± 1.43 c	75.15 ± 3.46 b	151.92 ± 9.12 a
*ZbUFGT*	36.74 ± 0.59 d	84.75 ± 3.92 b	103.24 ± 4.06 a	84.03 ± 1.41 c

S1–S4 represent the four gradual ripening stages of Z. bungeanum fruit: S1 (green stage), S2 (light red stage), S3 (red stage), and S4 (deep red stage). Different lowercase letters indicate significant differences among treatments according to one-way ANOVA test (*p* < 0.05).

## Data Availability

The Illumina raw sequencing profiles were submitted to the NCBI BioProject database under number PRJNA807160.
